# Paediatric cardiac surgery in Serbia – who are we closer to?

**DOI:** 10.1186/1749-8090-8-S1-P160

**Published:** 2013-09-11

**Authors:** M Stajevic

**Affiliations:** 1 Pediatric Cardiac Surgery, Mother and Child Health Institute of Serbia, Belgrade, Serbia

## Background

Department for Congenital Heart Surgery at the Mother and Child Health Institute of Serbia dates from 1982.

## Methods

The team consists of 2 surgeons, one resident, three anaesthesiologists and 2 intensivists. The number of operated cases at the Mother and Child Health Institute of Serbia is between 130 and 160 per year.

## Results

In 2003. four projects commenced: the arterial switch operation, Norwood I, redo surgery and valvular surgery. For arterial switch, after initial successes, came a series of deaths resulting in a high mortality of over 40% . The analysis of the risk factors showed that beside surgical misses, the reasons were various levels of motivation and dedication between different members of the surgical team. Only after reorganization the results changed dramatically: the mortality rate for ASO is now under 8%. The crucial identified risk factor was long anaesthesia induction and management of the immediate postoperative care. The results of treatment of HLHS with an overall mortality above 75% are still distant from the current standards. Inadequate preoperative management and late referrals are the main reasons for mortality. Children with valvular anomalies are dominantly treated with valve replacements. The univentricular heart patients have longer postoperative recovery, mortality varies between 12% and 16%. The results of the routine paediatric cardiac surgery are consistent with results of the major cited literature data.

ECMO and heart transplant are not available in our country. The lack of financial and donor support and the potentially low number of patients gravitating towards our institution question the cost benefit ratio.

## Conclusions

Compared to the surrounding countries, the provided services offered at our hospital can be regarded as cheaper, available and up to the standards of contemporary mid developed European countries.

